# 3-Methyl­amino-3-phenyl­propan-1-ol

**DOI:** 10.1107/S160053681203694X

**Published:** 2012-09-05

**Authors:** Wolfgang Frey, Mohammad M. Ibrahim, Basem F. Ali, Volker Jäger

**Affiliations:** a Universität Stuttgart, Institut für Organische Chemie, Pfaffenwaldring 55, D-70569 Stuttgart, Germany; b Department of Chemistry, Al al-Bayt University, Mafraq 25113, Jordan

## Abstract

The title compound, C_10_H_15_NO, is an amino alcohol with the hy­droxy group residing on the terminal C atom. Apart from the hy­droxy group and the phenyl ring, all non-H atoms are almost coplanar. In the crystal, classical O—H⋯N and N—H⋯O hydrogen bonds connect the mol­ecules into centrosymmetric dimers [*R*_2_^2^(12) descriptor] and tetra­meric units [*R*_4_^4^(8) descriptor] as ring motifs, consolidating a three-dimensional network.

## Related literature

For the syntheses of amino alcohols from isoxazolidines, isoxazolines and isoxazolinium salts, see: DeShong & Leginus, (1983 ▶); Henneböhle *et al.* (2004 ▶); Ibrahim (2009 ▶); Jäger & Buss, (1980 ▶); Jäger *et al.* (1985 ▶, 2010 ▶); Jäger & Colinas (2002 ▶); Lubell *et al.* (1991 ▶). For hydrogen-bond motifs see: Bernstein *et al.* (1995 ▶). For standard bond lengths, see: Allen *et al.* (1987 ▶).
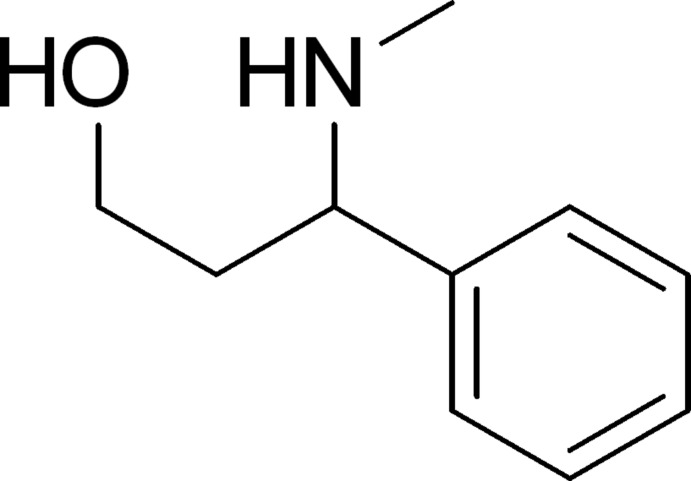


## Experimental

### 

#### Crystal data

C_10_H_15_NO
*M* *_r_* = 165.23
Monoclinic, 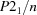

*a* = 5.9816 (8) Å
*b* = 23.8962 (19) Å
*c* = 7.4653 (8) Å
β = 111.119 (7)°
*V* = 995.40 (19) Å^3^
*Z* = 4
Cu *K*α radiation
μ = 0.56 mm^−1^
*T* = 293 K
0.80 × 0.50 × 0.20 mm

#### Data collection

Siemens P4 diffractometer
3535 measured reflections
1704 independent reflections
896 reflections with *I* > 2σ(*I*)
*R*_int_ = 0.087
3 standard reflections every 100 reflections intensity decay: 3%

#### Refinement

*R*[*F* ^2^ > 2σ(*F* ^2^)] = 0.071
*wR*(*F* ^2^) = 0.201
*S* = 1.04
1704 reflections
119 parameters
H atoms treated by a mixture of independent and constrained refinement
Δρ_max_ = 0.22 e Å^−3^
Δρ_min_ = −0.22 e Å^−3^

### 

Data collection: *XSCANS* (Bruker, 1996 ▶); cell refinement: *XSCANS*; data reduction: *XSCANS*; program(s) used to solve structure: *SHELXS97* (Sheldrick, 2008 ▶); program(s) used to refine structure: *SHELXL97* (Sheldrick, 2008 ▶); molecular graphics: *XP* in *SHELXTL-Plus* (Sheldrick, 2008 ▶); software used to prepare material for publication: *XP* in *SHELXTL-Plus*.

## Supplementary Material

Crystal structure: contains datablock(s) I, global. DOI: 10.1107/S160053681203694X/im2389sup1.cif

Structure factors: contains datablock(s) I. DOI: 10.1107/S160053681203694X/im2389Isup2.hkl

Supplementary material file. DOI: 10.1107/S160053681203694X/im2389Isup3.cml

Additional supplementary materials:  crystallographic information; 3D view; checkCIF report

## Figures and Tables

**Table 1:** Hydrogen-bond geometry (Å, °)

*D*—H⋯*A*	*D*—H	H⋯*A*	*D*⋯*A*	*D*—H⋯*A*
N1—H1*B*⋯O1^i^	1.02 (4)	2.06 (3)	3.023 (4)	157 (2)
O1—H1*A*⋯N1^ii^	1.12 (4)	1.70 (4)	2.815 (3)	176 (3)

Symmetry codes: (i) 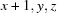; (ii) 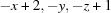.

**Fig. 1.**
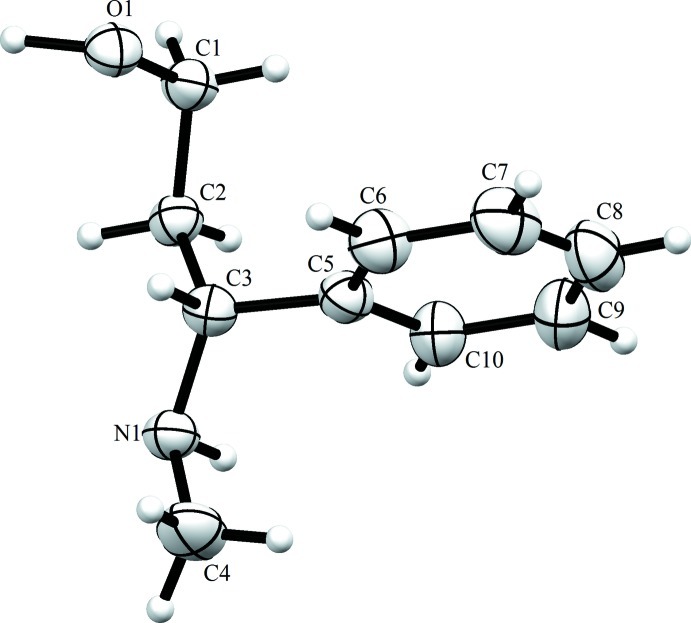
The molecular structure of I, with thermal ellipsoids drawn at the 30% probability level.

**Fig. 2.**
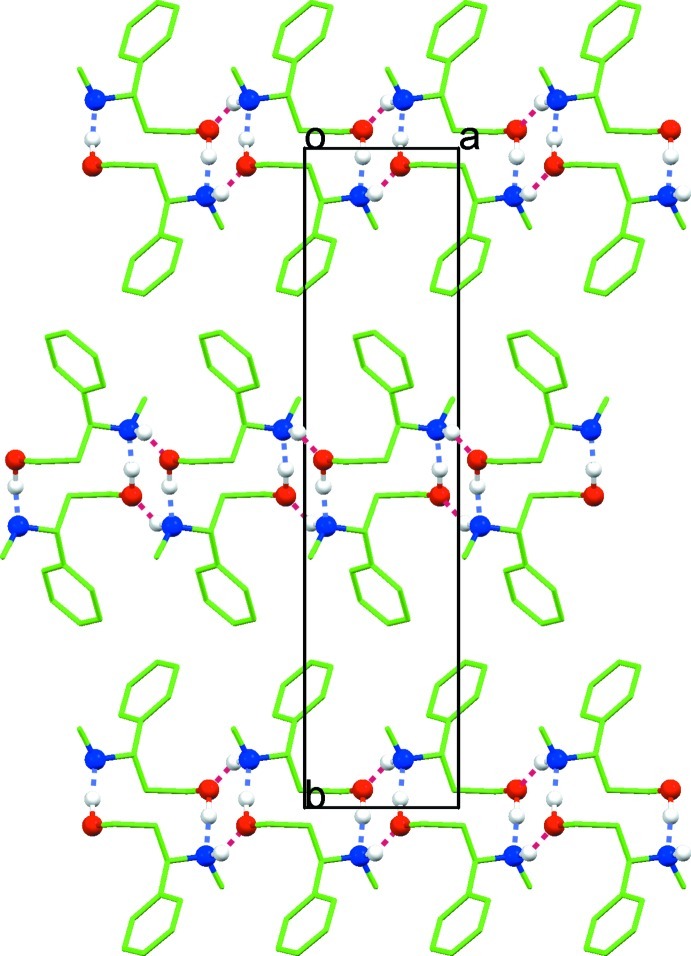
A view down c axis showing chains of hydrogen bonding molecules along a axis. The chains consist of alternating centrosymmetric dimers, with each dimer further interacting through the hydroxyl and amino groups with the adjacent one to form tetrameric units.

## supplementary crystallographic information

### Comment

Isoxazolidines, isoxazolines, and isoxazolinium salts are useful intermediates for syntheses of 1,3-amino alcohols by reduction with cleavage of the N–O bond (DeShong & Leginus, 1983; Jäger & Buss, 1980; Jäger *et al.*, 1985; Jäger & Colinas, 2002; Henneböhle *et al.*, 2004; Jäger *et al.*, 2010). The structures and conformations of previously synthesized amino alcohols were all assigned on the basis of analytical as well as IR, ^13^C and ^1^H NMR data. When the 2-methyl-3-phenylisoxazolidine-3-carbonitrile was heated to reflux with lithium aluminium hydride in ether (abs.), the title compound I was formed in good yield. The starting isoxazolidine had been obtained from the corresponding *N*-methyl-isoxazolinium salt by cyanide addition (Henneböhle *et al.*, 2004; Ibrahim, 2009; Jäger *et al.* 2010). The formation of the amino alcohol I was rationalized elsewhere (Ibrahim, 2009). The title compound I is already known from other routes (Lubell *et al.*, 1991), yet, the crystal structure of I has not been published so far. We herein report the synthesis and the crystal structure of I, along with the supramolecular motifs present in the crystal lattice.

The asymmetric unit of I consists of one amino alcohol molecule with bond distances and angles in the normal range (Allen *et al.*, 1987). The molecule, a primary alcohol and a secondary amine, adopts a planar zigzag-chain conformation (C1/C2/C3/N1/C4 almost coplanar), with both the hydroxy and the phenyl group being out-of-plane. The hydroxy and the phenyl group enclose dihedral angles of -60.3 (4)° and -63.0 (3)°, respectively, with the atoms of the carbon-chain (hydroxyl-O1/phenyl-C5-C1-C2-C3), see Fig. 1. In the crystal structure, molecules are hydrogen-bonded through the hydroxy groups as well as the amino groups (Table 1) giving rise to a three-dimensional network. The cooperative hydrogen bonds (alternating between hydroxy and amino groups) connect the molecules into chains down the crystallographic *a* axis (Fig. 2). These chains consist of alternating centrosymmetric dimers, with each dimer further interacting through the hydroxyl and amino groups with the adjacent one to form tetrameric units (Fig. 2). In terms of graph-set description, the hydrogen-bonded molecules might be described as forming two types of rings (Bernstein *et al.*, 1995), the centrosymmetric dimers being *R_2_^2^(12)* while *R_4_^4^(8)* represents the descriptor for the tetramer units. These interactions consolidate a three-dimensional network.

This amino alcohol conformation in the crystal found here is in contrast to the conformations elucidated in solution on the basis of IR dilution experiments and extensive collections of ^13^C and ^1^H NMR data, notably coupling constants and substituent increments - there intramolecular hydrogen bonds O—H···N prevail to form monomers with chair-like arrangements (Jäger & Buss, 1980).

### Experimental

A solution of 2-methyl-3-phenylisoxazolidine-3-carbonitrile (150 mg, 0.80 mmol) in anhydrous diethylether (5 ml) at 0°C was added to a suspension of LiAlH_4_ (61.0 mg, 1.6 mmol, 2 eq) in anhydrous diethylether (15 ml) and stirred for 30 min. The mixture was allowed to warm up to room temperature and stirred for 1 h. The reaction mixture was then heated to reflux for 12 h. The solution was cooled to r.t., then at 0°C with vigorous stirring. H_2_O (0.5 ml), 5% NaOH (0.5 ml), and H_2_O (1.5 ml) were added sequentially. The reaction mixture was extracted with ether (4 *x* 15 ml) and CH_2_Cl_2_ (2 *x* 10 ml). The organic layers were dried over Na_2_SO_4_. The solvent was evaporated *in vacuo* (5 mbar, 40°C) to afford the amino alcohol I in analytically and spectroscopically pure form as a colorless solid [100 mg, 84%, m.p. 56–57°C; lit. 56–57°C (Lubell *et al.*, 1991)]. Crystallization of the solid from ether afforded colorless crystals suitable for crystal structure determination. Analysis for C_10_H_15_NO, Calc.: C 72.69, H 9.15, N 8.48; Found: C 72.39, H 9.11, N 8.07.

### Refinement

Hydrogen atoms were located from the difference fourier map, but refined with fixed individual displacement parameters, using a riding model with d(C—H) ranging from 0.93 to 0.98 Å and U_iso_(H) = 1.2 U_eq_(C) or U_iso_(H) = 1.5 U_eq_(C_methyl_). In addition, the methyl group is allowed to rotate but not to tip. Hydrogen atoms attached to the hydroxy function and to the amino moiety are refined freely because of their relevance in hydrogen bonding.

### Crystal data

**Table:**

C_10_H_15_NO	*F*(000) = 360
*M**_r_* = 165.23	*D*_x_ = 1.103 Mg m^−^^3^
Monoclinic, *P*2_1_/*n*	Cu *K*α radiation, λ = 1.54178 Å
Hall symbol: -P 2yn	Cell parameters from 30 reflections
*a* = 5.9816 (8) Å	θ = 21.0–22.5°
*b* = 23.8962 (19) Å	µ = 0.56 mm^−^^1^
*c* = 7.4653 (8) Å	*T* = 293 K
β = 111.119 (7)°	Block, colourless
*V* = 995.40 (19) Å^3^	0.80 × 0.50 × 0.20 mm
*Z* = 4	

### Data collection

**Table:**

Siemens P4 diffractometer	*R*_int_ = 0.087
Radiation source: fine-focus sealed tube	θ_max_ = 67.5°, θ_min_ = 3.7°
Graphite monochromator	*h* = −7→7
ω scans	*k* = −28→28
3535 measured reflections	*l* = −8→8
1704 independent reflections	3 standard reflections every 100 reflections
896 reflections with *I* > 2σ(*I*)	intensity decay: 3%

### Refinement

**Table:**

Refinement on *F*^2^	Secondary atom site location: difference Fourier map
Least-squares matrix: full	Hydrogen site location: inferred from neighbouring sites
*R*[*F*^2^ > 2σ(*F*^2^)] = 0.071	H atoms treated by a mixture of independent and constrained refinement
*wR*(*F*^2^) = 0.201	*w* = 1/[σ^2^(*F*_o_^2^) + (0.0232*P*)^2^ + 0.9564*P*] where *P* = (*F*_o_^2^ + 2*F*_c_^2^)/3
*S* = 1.04	(Δ/σ)_max_ < 0.001
1704 reflections	Δρ_max_ = 0.22 e Å^−^^3^
119 parameters	Δρ_min_ = −0.22 e Å^−^^3^
0 restraints	Extinction correction: *SHELXL97* (Sheldrick, 2008), Fc^*^=kFc[1+0.001xFc^2^λ^3^/sin(2θ)]^-1/4^
Primary atom site location: structure-invariant direct methods	Extinction coefficient: 0.026 (2)

### Special details

**Table:**

Experimental. ^1^H NMR (500.2 MHz, MeOD): d = 1.82 (dddd, ^3^*J*_1a,2a _= 7.9 Hz, ^3^*J*_1b,2a_ = 5.6 Hz, ^2^*J*_2a,2b_ = 14.0 Hz, ^3^*J*_2a,3 _= 5.2 Hz, 1 H, 2-H_a_), 2.06 (dddd, ^3^*J*_1a,2b _= 6.2 Hz, ^3^*J*_1b,2b_ = 7.5 Hz, ^2^*J*_2a,2b_ = 14.2 Hz, ^3^*J*_2b,3 _= 5.8 Hz, 1 H, 2-H_b_), 2.18 (s, 3 H, NCH_3_), 3.42 (ddd, ^2^*J*_1a,1b _= 10.8 Hz, ^3^*J*_1a,2a_ = 8.1 Hz, ^3^*J*_1a,2b_ = 6.1 Hz, 1 H, 1-H_a_), 3.49 (ddd, ^2^*J*_1a,1b _= 8.0 Hz, ^3^*J*_1b,2a_ = 5.0 Hz, ^3^*J*_1b,2b_ = 6.8 Hz, 1 H, 1-H_b_), 3.65 (dd, ^3^*J*_2a,3 _= 8.3 Hz, ^3^*J*_2b,3_ = 6.0 Hz, 1 H, 3-H), 7.23-7.35 (m, 5 H, C_6_H_5_); ^13^C NMR (125.8 MHz, MeOD): d = 34.0 (q, NCH_3_), 40.7 (t, C-2), 60.4 (t, C-1), 63.7 (d, C-3), 128.2, 128.4, 129.6 (3 d, *o*-, *m*-, *p*-C of C_6_H_5_), 143.8 (s, *i*-C of C_6_H_5_).
Geometry. All e.s.d.'s (except the e.s.d. in the dihedral angle between two l.s. planes) are estimated using the full covariance matrix. The cell e.s.d.'s are taken into account individually in the estimation of e.s.d.'s in distances, angles and torsion angles; correlations between e.s.d.'s in cell parameters are only used when they are defined by crystal symmetry. An approximate (isotropic) treatment of cell e.s.d.'s is used for estimating e.s.d.'s involving l.s. planes.
Refinement. Refinement of *F*^2^ against ALL reflections. The weighted *R*-factor *wR* and goodness of fit *S* are based on *F*^2^, conventional *R*-factors *R* are based on *F*, with *F* set to zero for negative *F*^2^. The threshold expression of *F*^2^ > σ(*F*^2^) is used only for calculating *R*-factors(gt) *etc*. and is not relevant to the choice of reflections for refinement. *R*-factors based on *F*^2^ are statistically about twice as large as those based on *F*, and *R*- factors based on ALL data will be even larger.

### Fractional atomic coordinates and isotropic or equivalent isotropic displacement parameters (Å^2^)

**Table:**

	*x*	*y*	*z*	*U*_iso_*/*U*_eq_	
O1	0.6223 (4)	0.02730 (9)	0.2754 (3)	0.0633 (7)	
H1A	0.622 (7)	−0.0130 (18)	0.351 (6)	0.111 (14)*	
N1	1.3595 (5)	0.07300 (10)	0.5221 (4)	0.0610 (8)	
H1B	1.458 (6)	0.0688 (12)	0.436 (5)	0.063 (9)*	
C1	0.7636 (6)	0.02462 (14)	0.1583 (5)	0.0639 (9)	
H1C	0.7208	0.0555	0.0681	0.077*	
H1D	0.7277	−0.0099	0.0849	0.077*	
C2	1.0300 (5)	0.02701 (12)	0.2739 (4)	0.0564 (8)	
H2A	1.1168	0.0238	0.1868	0.068*	
H2B	1.0732	−0.0049	0.3599	0.068*	
C3	1.1102 (5)	0.08083 (12)	0.3929 (4)	0.0565 (8)	
H3	1.0121	0.0848	0.4727	0.068*	
C4	1.4610 (8)	0.11951 (15)	0.6516 (6)	0.0922 (14)	
H4A	1.4654	0.1522	0.5782	0.138*	
H4B	1.6207	0.1102	0.7351	0.138*	
H4C	1.3637	0.1268	0.7270	0.138*	
C5	1.0643 (6)	0.13176 (12)	0.2623 (5)	0.0603 (9)	
C6	0.8826 (7)	0.16881 (14)	0.2513 (6)	0.0790 (11)	
H6	0.7948	0.1640	0.3307	0.095*	
C7	0.8291 (8)	0.21376 (16)	0.1211 (7)	0.0936 (14)	
H7	0.7072	0.2387	0.1152	0.112*	
C8	0.9553 (9)	0.22067 (16)	0.0047 (7)	0.0925 (14)	
H8	0.9167	0.2496	−0.0844	0.111*	
C9	1.1390 (8)	0.18531 (15)	0.0176 (6)	0.0851 (12)	
H9	1.2292	0.1911	−0.0595	0.102*	
C10	1.1927 (7)	0.14055 (14)	0.1451 (5)	0.0737 (10)	
H10	1.3169	0.1163	0.1511	0.088*	

### Atomic displacement parameters (Å^2^)

**Table:**

	*U*^11^	*U*^22^	*U*^33^	*U*^12^	*U*^13^	*U*^23^
O1	0.0526 (13)	0.0690 (14)	0.0665 (15)	0.0047 (10)	0.0194 (11)	0.0030 (11)
N1	0.0552 (15)	0.0620 (16)	0.0583 (17)	−0.0054 (12)	0.0114 (14)	−0.0037 (12)
C1	0.0563 (18)	0.075 (2)	0.0562 (19)	−0.0061 (15)	0.0158 (16)	−0.0016 (15)
C2	0.0557 (17)	0.0585 (17)	0.0554 (18)	−0.0043 (13)	0.0206 (15)	−0.0044 (13)
C3	0.0547 (17)	0.0579 (17)	0.0563 (19)	0.0004 (13)	0.0193 (16)	−0.0015 (13)
C4	0.101 (3)	0.071 (2)	0.079 (3)	−0.015 (2)	0.001 (2)	−0.0142 (19)
C5	0.0563 (18)	0.0562 (17)	0.063 (2)	0.0016 (14)	0.0145 (16)	0.0007 (14)
C6	0.078 (2)	0.066 (2)	0.092 (3)	0.0102 (17)	0.030 (2)	−0.0017 (19)
C7	0.090 (3)	0.065 (2)	0.109 (4)	0.021 (2)	0.016 (3)	0.005 (2)
C8	0.114 (3)	0.067 (2)	0.082 (3)	0.009 (2)	0.019 (3)	0.011 (2)
C9	0.106 (3)	0.071 (2)	0.084 (3)	−0.001 (2)	0.040 (3)	0.0100 (19)
C10	0.082 (2)	0.068 (2)	0.073 (2)	0.0038 (17)	0.031 (2)	0.0077 (17)

### Geometric parameters (Å, º)

**Table:**

O1—C1	1.420 (4)	C4—H4B	0.9600
O1—H1A	1.12 (4)	C4—H4C	0.9600
N1—C4	1.454 (4)	C5—C10	1.373 (5)
N1—C3	1.468 (4)	C5—C6	1.381 (4)
N1—H1B	1.02 (4)	C6—C7	1.406 (5)
C1—C2	1.516 (4)	C6—H6	0.9300
C1—H1C	0.9700	C7—C8	1.351 (6)
C1—H1D	0.9700	C7—H7	0.9300
C2—C3	1.538 (4)	C8—C9	1.362 (6)
C2—H2A	0.9700	C8—H8	0.9300
C2—H2B	0.9700	C9—C10	1.390 (5)
C3—C5	1.521 (4)	C9—H9	0.9300
C3—H3	0.9800	C10—H10	0.9300
C4—H4A	0.9600		
			
C1—O1—H1A	112 (2)	N1—C4—H4B	109.5
C4—N1—C3	114.9 (3)	H4A—C4—H4B	109.5
C4—N1—H1B	107.1 (17)	N1—C4—H4C	109.5
C3—N1—H1B	106.3 (18)	H4A—C4—H4C	109.5
O1—C1—C2	112.6 (3)	H4B—C4—H4C	109.5
O1—C1—H1C	109.1	C10—C5—C6	118.2 (3)
C2—C1—H1C	109.1	C10—C5—C3	121.2 (3)
O1—C1—H1D	109.1	C6—C5—C3	120.5 (3)
C2—C1—H1D	109.1	C5—C6—C7	120.6 (4)
H1C—C1—H1D	107.8	C5—C6—H6	119.7
C1—C2—C3	113.9 (3)	C7—C6—H6	119.7
C1—C2—H2A	108.8	C8—C7—C6	119.8 (4)
C3—C2—H2A	108.8	C8—C7—H7	120.1
C1—C2—H2B	108.8	C6—C7—H7	120.1
C3—C2—H2B	108.8	C7—C8—C9	120.2 (4)
H2A—C2—H2B	107.7	C7—C8—H8	119.9
N1—C3—C5	115.4 (2)	C9—C8—H8	119.9
N1—C3—C2	107.7 (2)	C8—C9—C10	120.5 (4)
C5—C3—C2	110.6 (2)	C8—C9—H9	119.8
N1—C3—H3	107.6	C10—C9—H9	119.8
C5—C3—H3	107.6	C5—C10—C9	120.7 (4)
C2—C3—H3	107.6	C5—C10—H10	119.7
N1—C4—H4A	109.5	C9—C10—H10	119.7
			
O1—C1—C2—C3	−60.3 (4)	C10—C5—C6—C7	0.9 (5)
C4—N1—C3—C5	58.3 (4)	C3—C5—C6—C7	−175.6 (3)
C4—N1—C3—C2	−177.6 (3)	C5—C6—C7—C8	0.4 (6)
C1—C2—C3—N1	169.9 (3)	C6—C7—C8—C9	−2.1 (7)
C1—C2—C3—C5	−63.0 (3)	C7—C8—C9—C10	2.5 (7)
N1—C3—C5—C10	54.1 (4)	C6—C5—C10—C9	−0.6 (5)
C2—C3—C5—C10	−68.5 (4)	C3—C5—C10—C9	175.9 (3)
N1—C3—C5—C6	−129.5 (3)	C8—C9—C10—C5	−1.1 (6)
C2—C3—C5—C6	107.9 (3)		

### Hydrogen-bond geometry (Å, º)

**Table:**

*D*—H···*A*	*D*—H	H···*A*	*D*···*A*	*D*—H···*A*
N1—H1*B*···O1^i^	1.02 (4)	2.06 (3)	3.023 (4)	157 (2)
O1—H1*A*···N1^ii^	1.12 (4)	1.70 (4)	2.815 (3)	176 (3)

Symmetry codes: (i) *x*+1, *y*, *z*; (ii) −*x*+2, −*y*, −*z*+1.

## References

[bb1] Allen, F. H., Kennard, O., Watson, D. G., Brammer, L., Orpen, A. G. & Taylor, R. (1987). *J. Chem. Soc. Perkin Trans. 2* pp. S1–19.

[bb2] Bernstein, J., Davis, R. E., Shimoni, L. & Chang, N.-L. (1995). *Angew. Chem. Int. Ed. Engl.* **34**, 1555-1573.

[bb3] Bruker (1996). *XSCANS* Bruker AXS Inc., Madison, Wisconsin, USA.

[bb4] DeShong, P. & Leginus, J. M. (1983). *J. Am. Chem. Soc.* **105**, 1686-1688.

[bb5] Henneböhle, M., Le Roy, P.-Y., Hein, M., Ehrler, R. & Jäger, V. (2004). *Z. Naturforsch. Teil B*, **59**, 451– 467.

[bb6] Ibrahim, M. M. (2009). Dissertation, Universität Stuttgart, Germany.

[bb7] Jäger, V. & Buss, V. (1980). *Liebigs Ann. Chem.* pp. 101–121.

[bb8] Jäger, V. & Colinas, P. (2002). *Synthetic Applications of 1,3-Dipolar Cycloaddition Chemistry Toward Heterocycles and Natural Products, The Chemistry of Heterocyclic Compounds*, edited by A. Padwa & W. H. Pearson, pp. 361–472. New York: Wiley.

[bb9] Jäger, V., Frey, W., Bathich, Y., Shiva, S., Ibrahim, M., Henneböhle, M., LeRoy, P. Y. & Imerhasan, M. (2010). *Z. Naturforsch. Teil B*, **65b**, 821–832.

[bb10] Jäger, V., Müller, I. & Paulus, E. F. (1985). *Tetrahedron Lett.* **26**, 2997-3000.

[bb11] Lubell, W. D., Kitamura, M. & Noyori, R. (1991). *Tetrahedron Asymmetry*, **2**, 543-554.

[bb12] Sheldrick, G. M. (2008). *Acta Cryst.* A**64**, 112–122. 10.1107/S0108767307043930 18156677

